# Laparoscopic Abdominal Cerclage as an Effective Option for Refractory Cervical Insufficiency: A Case Report

**DOI:** 10.7759/cureus.101329

**Published:** 2026-01-12

**Authors:** Sara Paiva, Hugo Barros, Luísa Machado, Ricardo Santos, Pedro Oliveira

**Affiliations:** 1 Department of Obstetrics and Gynaecology, Unidade Local de Saúde do Alto Ave, Guimarães, PRT; 2 Center for Health Technology and Services Research, Faculdade de Medicina da Universidade do Porto, Porto, PRT

**Keywords:** cervical insufficiency, laparoscopic cerclage, pregnancy, preterm birth, transabdominal cerclage

## Abstract

Cervical insufficiency is an important cause of preterm birth and fetal loss and is traditionally treated by transvaginal cervical cerclage. However, when this technique has failed or is not feasible, transabdominal cerclage may be performed, with the laparoscopic approach emerging as the preferred option. We report the case of a 35-year-old woman with recurrent second-trimester loss and a prior failed transvaginal cerclage, who underwent laparoscopic abdominal cerclage at nine weeks’ gestation. The procedure was uncomplicated, and the pregnancy ensued without major events. An elective caesarean section was performed at 37 weeks’ gestation, resulting in the delivery of a 2765g newborn with an Apgar score of 9/10. This case illustrates the effectiveness of laparoscopic abdominal cerclage as a safe, reproducible, and highly effective option for women with refractory cervical insufficiency.

## Introduction

Cervical insufficiency is an important cause of preterm birth and fetal loss, complicating up to 1% of pregnancies. It is traditionally treated by transvaginal cervical cerclage, which involves placing a strong suture around the cervix, via the vagina, and tightening it to keep the cervix closed. It is then usually removed at around 37 weeks of gestation to allow delivery [1]. However, in cases where this technique has failed or when it is not feasible due to a short length or a scarred cervix, the cerclage may be placed transabdominally. This approach provides added integrity to the cervix, as it is placed higher up at the level of the cervico-isthmic junction, with less risk of caudal suture migration as the uterus enlarges. In addition, the absence of a foreign body in the vagina may reduce the risk of ascending infection. Growing evidence supports abdominal cerclage as being superior to repeated vaginal cerclage in reducing the risk of early preterm birth and fetal loss in women with previous failed vaginal cerclage [1,2]. This can be achieved via either open abdominal or laparoscopic approaches, with the latter emerging as the preferred option, as it provides similar or superior gestational and neonatal outcomes while offering the benefits of minimally invasive surgery [2-4]. There is no consensus regarding the optimal timing for the procedure. It can be safely performed both before and after conception, most commonly in the first trimester of pregnancy, with no significant differences in live birth rates [5,6].

Furthermore, a study by Vousden et al. [7] found that placement of a transabdominal cerclage before conception does not negatively affect fertility. Two types of tapes have been described, double-armed Mersilene® tape and Prolene® suture, but no longitudinal study comparing them has been identified in the literature [8]. Straightened needles can be used, as they allow more precise needle guidance. The suture may be inserted in either direction, passing at the level of the uterine isthmus medial to the uterine vessels; alternatively, it can be inserted laterally to the uterine vessels and above the ureters at the level of the uterine isthmus, above the uterosacral ligament as described in the modified method by Shin et al. [9] The knot can be placed in three different positions: anterior, posterior, or intravaginal. The first has the advantage of avoiding the risk of adhesions in the Douglas pouch and can be easily removed in laparoscopy, but may increase the risk of erosion into the bladder. In post-conceptional cerclage, it is preferably placed anteriorly, while a posterior knot is recommended in pre-conceptional cerclage [8]. Patients with an abdominal cerclage require a caesarean delivery, performed between 37 and 39 weeks’ gestation, electively during which the suture can be removed or left in place if a future pregnancy is intended [2]. A preliminary version of the following clinical case was previously presented as a poster at the ESGE 31st Annual Congress of the European Society for Gynaecological Endoscopy, October 2022.

## Case presentation

**Figure 1:**
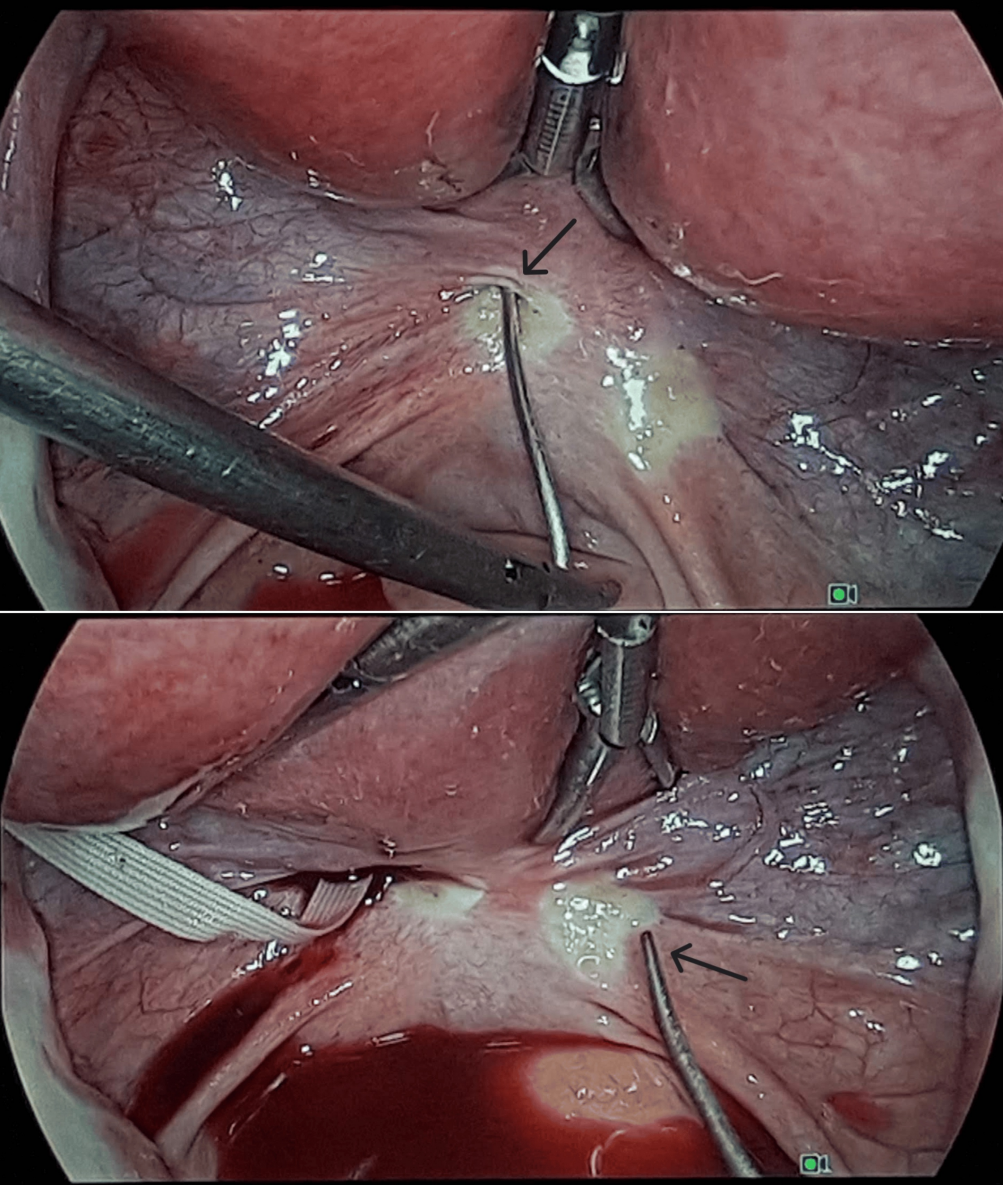
Insertion of a straightened needle of the 5-mm Mersilene® tape through the posterior aspect of the cervical isthmus, above the insertion point of the left uterosacral ligament (above). The same procedure was performed contralaterally (below).

A 35-year-old female, gravida 2, para 0, with both pregnancies following frozen embryo transfer, was referred to our centre for evaluation of cervical insufficiency. The patient had a history of medical termination of pregnancy due to preterm premature rupture of membranes at 18 weeks’ gestation. As fetal autopsy and cytogenetic testing revealed no anomalies, and thrombophilia screening, specifically lupus anticoagulant, anticardiolipin antibodies (IgG and IgM), and anti-β2 glycoprotein I antibodies (IgG and IgM), as well as bacteriological tests were negative, a prophylactic vaginal cerclage was placed at 14 weeks in her second pregnancy. However, at 22 weeks, she was admitted with mild pelvic pain associated with protruding membranes and an almost fully dilated cervix; the suture was therefore removed, leading to fetal delivery. She subsequently conceived spontaneously, and a laparoscopic cerclage was proposed and performed at nine weeks under general anaesthesia. No vaginal instrumentation was used. The vesicouterine peritoneum was dissected transversely using bipolar energy. A double-armed 5-mm Mersilene® tape was prepared by straightening the attached curved needles, and the needle tip was placed against the posterior aspect of the cervical isthmus, above the insertion point of the ipsilateral uterosacral ligament, and brought out anteriorly, pulling the ligature with it. The procedure was repeated on the contralateral side (Figure 1).

**Figure 2:**
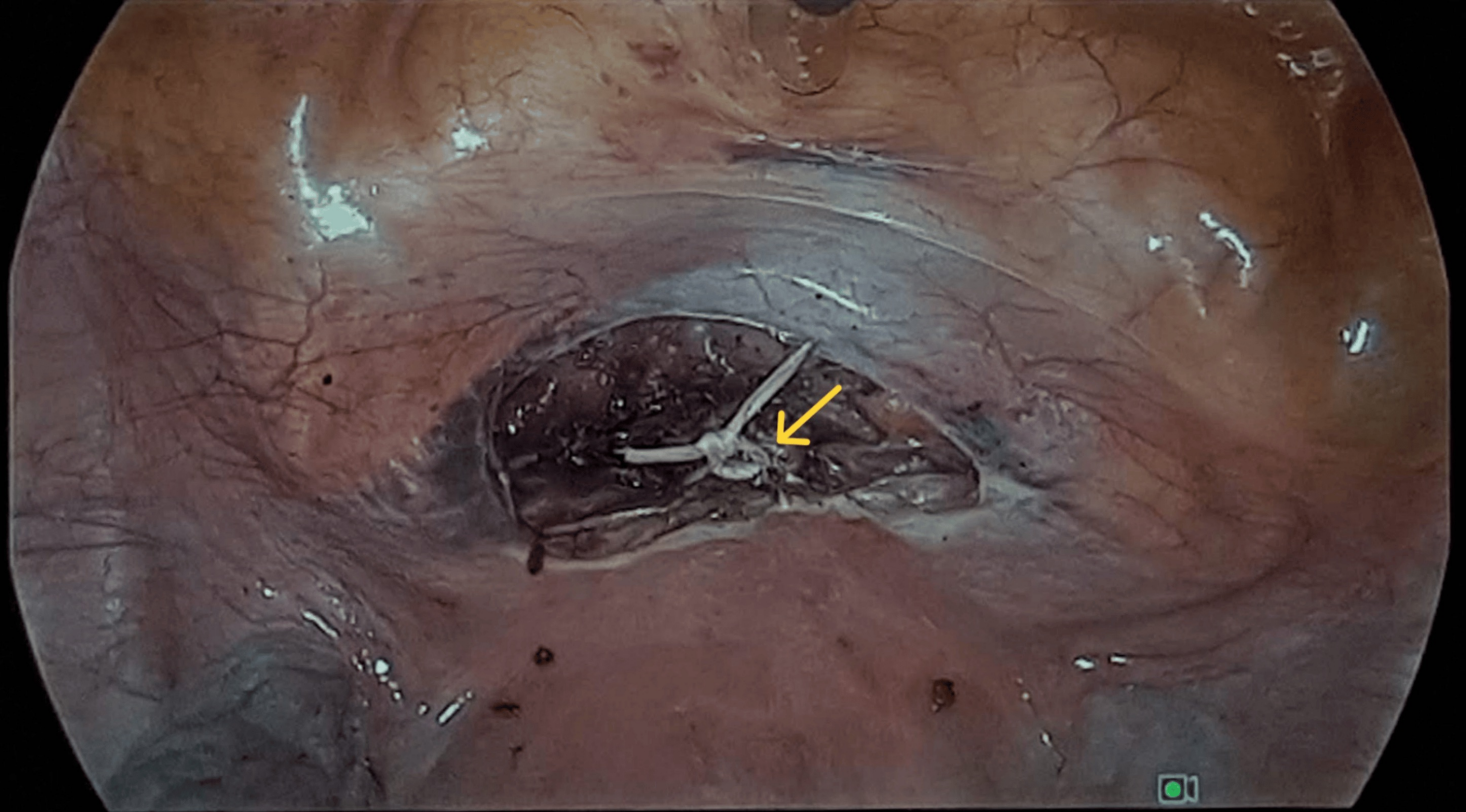
Cerclage knot placed anterior to the cervix.

The ligature ends were cut to release the needles, and the ends were tied tightly anteriorly (Figure 2). Reperitonealisation was then carried out. 

No tocolytic agents were used intraoperatively. Fetal cardiac activity was confirmed before and after the procedure. The procedure was uncomplicated, and the patient was discharged the following day. The pregnancy was then followed up in our centre, without major events. A caesarean section was undertaken at 37 weeks’ gestation, and a 2765g newborn was delivered with an Apgar score of 9/10. The cerclage was left in situ, and the postoperative course was uneventful.

## Discussion

Abdominal cerclage is increasingly being considered after a single failed vaginal cerclage, with accumulating evidence indicating that it may provide more effective outcomes than repeating a vaginal cerclage [2]. Our case illustrates the effectiveness of laparoscopic abdominal cerclage in a patient with recurrent second-trimester loss and a previous failed transvaginal cerclage. The minimally invasive approach allows reliable placement at the cervico-isthmic junction, with shorter recovery times and fewer complications [2]. Overall, reported operative complications are uncommon and depend mainly on the operator’s skill and expertise [8]. Although patients with an abdominal cerclage require delivery by caesarean section, the benefit regarding fetal survival outweighs this limitation, particularly in women with an otherwise poor prognosis. Another concern is mid-trimester pregnancy loss or the need for mid-trimester termination. Nevertheless, laparoscopic removal of the suture has been described, allowing vaginal delivery, with subsequent replacement of the cerclage [10]. In our patient, early pregnancy placement was uncomplicated and resulted in a term live birth, supporting the favourable obstetric and neonatal outcomes reported in the literature and thereby reinforcing this technique as a safe, reproducible and highly effective option for women with refractory cervical insufficiency. Notably, this was the first abdominal cerclage performed in our department and, to our knowledge, the first case reported in Portugal, underscoring the relevance of sharing our experience.

## Conclusions

Laparoscopic abdominal cerclage may be considered as a first-line option in patients with a prior failed vaginal cerclage, as it offers similar or improved obstetric and neonatal outcomes compared with alternative approaches, in addition to the benefits of minimally invasive surgery. However, further studies are needed to better define the optimal timing of the procedure and to refine the operative technique.
